# B-cell Lymphoblastic Lymphoma of the Paranasal Sinuses: A Case Study of a Rare Clinical Entity

**DOI:** 10.7759/cureus.31565

**Published:** 2022-11-16

**Authors:** Brian P Anderson, Christopher M Metz

**Affiliations:** 1 Otolaryngology - Head and Neck Surgery, Ascension Macomb-Oakland, Detroit, USA; 2 Otolaryngology - Head and Neck Surgery, Beaumont Hospital, Dearborn, USA

**Keywords:** acute sinusitis, non hodgkin's lymphoma, b lymphoblastic lymphoma, primary sinonasal lymphoma, paranasal sinus neoplasm

## Abstract

Lymphoblastic lymphoma is a rare and aggressive form of non-Hodgkin lymphoma (NHL). The tumor can derive from T-cell or B-cell and is clinically similar to acute lymphoblastic leukemia with minimal to no bone marrow involvement distinguishing the two. We present a rare case of lymphoblastic lymphoma originating from the paranasal sinuses. A 40-year-old male presented to the emergency department and was diagnosed with right-sided acute sinusitis complicated by pre-septal cellulitis. After failing medical management, he underwent endoscopic sinus surgery. Pathologic analysis of nasal contents stained for CD79a, CD34, and PAX5, suggesting a B-cell lymphoblastic lymphoma (B-LBL). He was referred to hematology-oncology and was treated with hyperfractionated cyclophosphamide, vincristine, Adriamycin, dexamethasone (Hyper-CVAD). Short-term follow-up has thus far demonstrated near-complete resolution of the tumor. Non-Hodgkin lymphomas of the paranasal sinuses are rare, and B-cell lymphoblastic lymphomas comprise just 0.3% of adults with NHL. Immunohistochemical phenotyping for B-LBL is typically positive for B-cell markers CD19, CD20, CD22 and CD79a. Classic treatment involves a chemotherapy regimen of Hyper-CVAD with an overall survival rate of 66%. B-cell lymphoblastic lymphoma is rarely reported in the paranasal sinuses. A thorough history and physical exam along with a complete workup, including biopsies with histopathological and immunohistochemical analysis, needs to be obtained. Little is known about its prognosis when the primary site is within the paranasal sinuses, and therefore, patients need prompt and aggressive treatment when the diagnosis is made.

## Introduction

This article was previously presented as a poster presentation at the AAO-HNSF 2022 Annual Meeting & OTO Experience on September 12, 2022. 

Lymphomas are malignant neoplasms of lymphocytes and their precursor cells. They account for 12% to 15% of head and neck malignancies, 75% of which present as nodal diseases [1]. A sinonasal lymphoma is a rare form of presentation, accounting for < 1% of all head and neck cancers [2]. Lymphomas are broadly divided into Hodgkin lymphoma (HL) and non-Hodgkin lymphoma (NHL). Hodgkin lymphoma is rarer, accounting for 10% of all lymphomas, compared to NHL, which makes up the remaining 90% [3]. On histology, HL is characterized by Reed-Sternberg cells, tumor giant cells that are bilobed or binucleate, having the colloquial appearance of “owl eyes” [4]. Non-Hodgkin lymphomas, in contrast, originate mainly from B-cells, but may also derive from T-cells or natural killer cells.

Extranodal origins of NHL in the head and neck region are commonly seen in the tonsils, nasopharynx, oropharynx, thyroid, salivary gland or sinuses, accounting for up to 15% to 20% of all large cell lymphomas. Predominant precursor cells in NHL of the paranasal sinuses and the nasal cavity classically vary based on certain demographics. In Asian and South American populations, sinonasal NHLs are predominately T-cell or natural killer cells in origin. In Western populations, sinonasal NHLs are predominately diffuse large B-cell lymphoma (DLBCL), the majority of which present within the maxillary sinus [1]. We report a case of a rare type of NHL, B-cell lymphoblastic lymphoma (B-LBL), of the paranasal sinus where the primary presenting symptoms were acute sinusitis with pre-septal cellulitis.

## Case presentation

A 40-year-old African American male presented to the Emergency Department with seven days of right-sided headache, purulent nasal drainage, and moderate ocular pain. The patient had a medical history significant for allergic rhinitis and chronic sinusitis. His surgical history was non-contributory. The patient’s social history included a 40-pack-year smoking history, cocaine use, marijuana use, and moderate alcohol use.

A head and neck physical exam revealed right-sided proptosis with swelling and erythema over the right upper eyelid along with pain with extraocular movement. Examination of the nose demonstrated right-sided purulent drainage, swollen and boggy turbinates, polypoid-like tissue nearly at the vestibule of the right nares, and swelling of the right submandibular area without evidence of erythema or fluctuance. Otologic exam showed a right-sided serous effusion of the middle ear.

**Figure 1:**
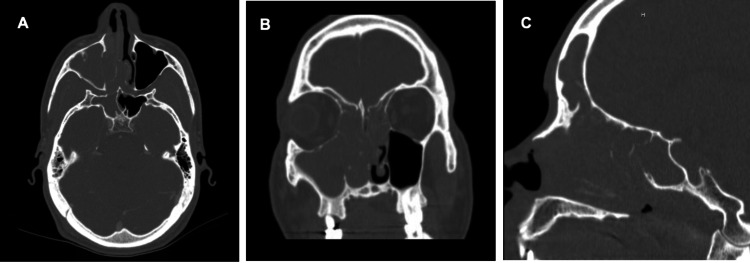
Computed tomography (CT) without contrast (A) Axial, (B) coronal, and (C) sagittal computed tomography (CT) without contrast showing a mass-like lesion occupying the paranasal sinuses, particularly involving the right maxillary, ethmoid, frontal and sphenoid sinus with extension into the right nasopharynx and left ethmoid sinus

A computerized tomography (CT) scan of the paranasal sinus without contrast was obtained which suggested extensive right-sided sinus disease involving the right maxillary, frontal, ethmoid and sphenoid sinuses with complete opacification, loss of bony septate, and erosion of the medial wall of the orbit. The left sphenoid sinus was also opacified and a polypoid lesion was seen in the inferior portion of the left maxillary sinus. Additionally, fluid was seen in the right mastoid air cells and right middle ear cavity (Figure 1).

These findings were concerning for significant acute sinusitis, possibly due to a fungal infection, along with possible periostitis or osteomyelitis of the medial wall of the orbit.

The patient was diagnosed with acute sinusitis complicated by preseptal cellulitis. He was placed on intravenous antibiotics per the infectious disease team. He was found to have minimal improvement with medical management. Treatment options were discussed with the patient and he elected for endoscopic sinus surgery with stereotactic navigation assistance. The patient underwent comprehensive endoscopic sinus surgery. Purulent polypoid tissue was noted in all sinuses with a heavy concentration in the patient’s right maxillary and ethmoid sinuses. The lamina papyracea was dehiscent on the patient’s right. Cultures were taken of the ethmoid sinus contents on the right, where the highest concentration of purulent material existed. Microdebrider contents were separated by side and sent for pathologic analysis.

The patient had an unremarkable, reassuring post-operative course. His ocular pain and headaches completely resolved. The patient reported relief of nasal obstruction and he was discharged home on post-operative date #2 to follow up-outpatient for debridement.

**Figure 2:**
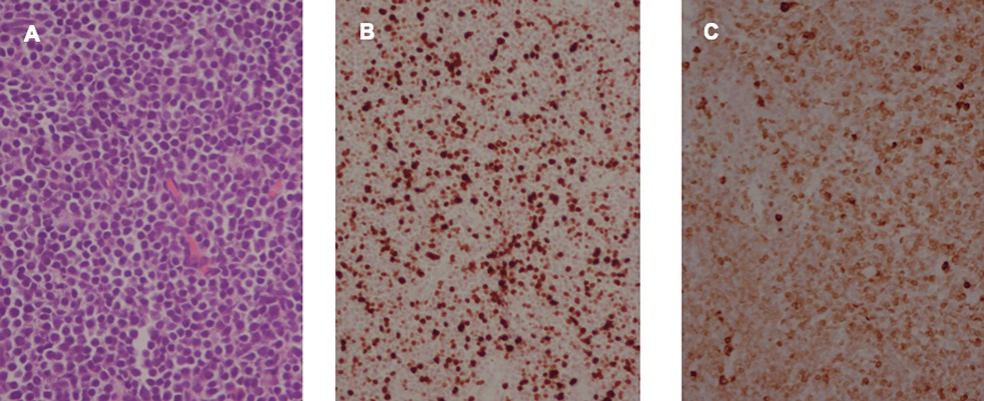
Pathological analysis of sinus contents A) Histologic examination (hematoxylin-eosin stain) of nasal contents with diffuse lymphocytic infiltration, 40X magnification. (B) Immunohistochemical stain of sinus contents is positive for Ki-67, 40X magnification. (C) Immunohistochemical stain of sinus contents is positive for CD79a, 40X magnification.

Sinus contents were sent to pathology for analysis. Histomorphology showed a diffuse, monotonous infiltrate of blastic cells with regular nuclear contours, delicate chromatin, scant cytoplasm, and very high mitotic activity. Immunohistochemical analysis demonstrated staining positive for CD79a, CD43, CD34, and PAX5, and weakly for TdT. The contents stained negative for CD10, CD20, CD23, CD138, or myeloperoxidase. Bone marrow biopsy was negative for any malignant cells. These findings were consistent with a B-cell lymphoblastic lymphoma (Figure 2).

The patient underwent imaging for staging. CT Chest, Abdomen, and Pelvis were unremarkable. Positron emission tomography scan using F-18 flourodeoxyglucose (FDG-PET) was performed which demonstrated FDG avid soft-tissue density within the right maxillary sinuses, right submandibular gland, and mildly FDG avid soft-tissue density within the bilateral cervical chain lymph nodes extending to the supraclavicular region. The patient was referred to oncology and he began chemotherapy promptly. Two-month FDG-PET scan follow-up demonstrated near complete resolution of FDG-uptake, indicating acute local-regional control of the disease.

## Discussion

Lymphomas are a diverse group of malignant neoplasms from various lymphocytic precursor cells, including natural killer cells, T-cells and B-cells. Lymphomas of the head and neck are common, but only < 1% present as primary sinonasal lymphoma [2]. However, the paranasal sinus has been shown to have worse overall survival compared to other more common head and neck subsites, such as Waldeyer’s Ring [5]. Prognosis depends on the cellular subtype, with overall survival ranging from 20-55% [6-8].

Lymphoblastic lymphoma is a rare subtype of NHL that is further subdivided into T-cell lineage (90%) and B-cell lineage (10%) [8]. Lymphoblastic lymphoma is histopathologically similar to B-cell acute lymphoblastic leukemia (B-ALL), and it is made distinct from B-ALL by primarily involving lymph nodes or extranodal sites and demonstrating minimal to no bone marrow involvement [9]. Overall, lymphoblastic lymphoma accounts for 2% of all NHL, and B-cell lymphoblastic lymphoma (B-LBL) comprises just 0.3% of adult NHL [10]. B-cell lymphoblastic lymphoma typically presents at extranodal sites, and the most common locations include the skin, bone and mediastinum [11]. Immunohistochemical phenotyping for B-LBL is typically positive for B-cell markers CD19, CD20, CD22 and CD79a. They may also be positive for CD10, CD24, PAX5, and TdT. Commonly, lymphoma can spread to the central nervous system, and so, spinal fluid needs to be analyzed. Finally, bone marrow biopsies need to be performed to distinguish B-LBL from B-ALL.

Treatment for B-LBL has varied over the years, with no optimal standard therapy. Classically, a chemotherapy regimen of hyperfractionated cyclophosphamide, vincristine, Adriamycin, and dexamethasone (Hyper-CVAD) alternating with high doses of methotrexate and cytarabine has been used as treatment for B-LBL with promising results. A study from M.D. Anderson Cancer Center has demonstrated a response rate of 91% with an overall survival rate of 66% [12]. However, modifications to current treatment protocols and newer treatment regimens are showing promise for even better overall survival [13].

## Conclusions

B-cell lymphoblastic lymphoma is a rare and aggressive form of non-Hodgkin lymphoma that needs prompt and thorough workup for survival. A thorough history and physical exam along with a complete workup, including biopsies with histopathological and immunohistochemical analysis, needs to be obtained. Lymphoblastic lymphoma is an aggressive form of NHL, but little is known about its prognosis when the primary site is within the paranasal sinuses, and therefore, patients need prompt and aggressive treatment when the diagnosis is made.
